# Narrowing the spread in CMIP5 model projections of air-sea CO_2_ fluxes

**DOI:** 10.1038/srep37548

**Published:** 2016-11-28

**Authors:** Lei Wang, Jianbin Huang, Yong Luo, Zongci Zhao

**Affiliations:** 1 Ministry of Education Key Laboratory for Earth System Modeling, Center for Earth System Science, and Joint Center for Global Change Studies (JCGCS), Tsinghua University, Beijing 100084, China

## Abstract

Large spread appears in the projection of air-sea CO_2_ fluxes using the latest simulations from the Coupled Model Intercomparison Project Phase 5 (CMIP5). Here, two methods are applied to narrow this spread in 13 CMIP5 models. One method involves model selection based on the ability of models to reproduce the observed air-sea CO_2_ fluxes from 1980 to 2005. The other method involves constrained estimation based on the strong relationship between the historical and future air-sea CO_2_ fluxes. The estimated spread of the projected air-sea CO_2_ fluxes is effectively reduced by using these two approaches. These two approaches also show great agreement in the global ocean and three regional oceans of the equatorial Pacific Ocean, the North Atlantic Ocean and the Southern Ocean, including the average state and evolution characteristics. Based on the projections of the two approaches, the global ocean carbon uptake will increase in the first half of the 21^st^ century then remain relatively stable and is projected to be 3.68–4.57 PgC/yr at the end of 21^st^ century. The projections indicate that the increase in the CO_2_ uptake by the oceans will cease at the year of approximately 2070.

The global increase in mean surface temperature since the 1950 s is very likely attributable to the increase in anthropogenic greenhouse gas concentrations and other human forces. The ocean has absorbed approximately 30% of the anthropogenic CO_2_ emissions from fossil fuel burning, cement production and land use changes since the industrial revolution^1^,^2^ and, between the industrial revolution (beginning in 1750) and 2011, has accumulated 155 ± 30 PgC^3^. Because the ocean is a large carbon pool and an important carbon sink, the exchange of CO_2_ fluxes between the air and ocean greatly influences the atmospheric CO_2_ concentration and further affects the air temperature.

The global ocean overall acts as a large carbon sink^4^,^5^,^6^,^7^, and the contribution from different oceanic regions actually varies. In contrast to the overall carbon sink nature of the global ocean, the equatorial Pacific Ocean is a major atmospheric carbon source^8^,^9^,^10^,^11^, which emits approximately 0.44 ± 0.14 PgC/yr^10^,^12^,^13^. This large source may due to the upwelling of dissolved inorganic carbon (DIC) from the deep ocean^14^,^15^. Unlike the equatorial Pacific Ocean, the North Atlantic Ocean is an important atmospheric CO_2_ sink. The North Atlantic represents only 13% of the total ocean area but absorbs approximately one-third of the total uptake of the global ocean^16^,^17^,^18^. Additionally, the Southern Ocean is a major atmospheric CO_2_ sink, contributing nearly half of the global ocean uptake of anthropogenic CO_2_^19^,^20^, and it is considered a critical component of the deglacial CO_2_ rise^21^,^22^,^23^. Because of the critical role of the ocean in the global carbon cycle, the future carbon uptake of the global ocean and three regional oceans, i.e., the equatorial Pacific Ocean, North Atlantic Ocean and Southern Ocean, will undoubtedly affect the rising CO_2_ concentration and temperature pathways.

Numerical models are widely used to diagnose and project changes in climate variables. Especially in the latest fifth phase of the Coupled Model Intercomparison Project (CMIP5), most of the models are earth system models (ESM), which couple the carbon cycle processes among the atmosphere, land and ocean^24^. ESMs can be driven directly by emissions, and the ocean carbon can interact with the atmospheric carbon, similar to the real natural processes. Thus, the atmospheric CO_2_ concentration is calculated by the models rather than being specified^25^,^26^. Therefore, the ESMs provide a useful way to simulate and project air-sea CO_2_ fluxes.

However, a large spread exists in the air-sea CO_2_ fluxes simulated by the ESMs^27^,^28^,^29^,^30^, and this variability may derive from the various resolutions, climate sensitivities and carbon components among the ESMs. In previous research, the range of air-sea CO_2_ fluxes simulated by the 11 models form Coupled Climate Carbon Cycle Model Intercomparison Project (C^4^MIP, which is a model intercomparison project for global climate models that include an interactive carbon cycle) was between 1.4 PgC/yr and 3 PgC/yr in 2000 and between 3.8 PgC/yr and 10 PgC/yr in 2100^30^. Additionally, 11 CMIP5 models driven by carbon emissions projected an ocean cumulative carbon uptake of 412–649 PgC in 2100^28^. The large spread in the air-sea CO_2_ flux estimates could be an additional source for climate projection uncertainty^28^. Hence, the air-sea CO_2_ flux simulation spread needs to be narrowed to reduce the uncertainty in future climate projections. Kessler and Tjiputra^31^ have proposed that the air-sea CO_2_ fluxes in the Southern Ocean could be used to constrain the future global ocean carbon uptake uncertainty. In this paper, two other methods are used to narrow the projected spread of air-sea CO_2_ fluxes.

The structure of the paper is as follows. The observation-based and model data used in this research are introduced first, and the two methods and results are described in section 2. A summary and discussion is presented in the last part.

## Data

In total, 13 CMIP5 models from 11 affiliations are used in this research (Table 1). The outputs of ESM runs that were forced by CO_2_ emissions and that fully coupled the carbon cycle between the atmosphere and ocean are selected to project the air-sea CO_2_ fluxes. The future climate projected in the ESM runs is under the representative concentration pathways 8.5 (RCP8.5). The model resolutions vary, and most of the durations extend from 1850 to 2005 for “esmhistorical” runs and from 2006 to 2100 for “esmrcp85” runs.

Observations are also needed to evaluate and constrain the performance of the models. Direct observations of air-sea CO_2_ fluxes are mainly provided by ships and buoys. Because of the difficulties in a wide and intensive observation, directly observed data are relatively sparse and lack of continuity. To make up for the lack of observations in time and space, researchers use various strategies to estimate the air-sea CO_2_ fluxes. Estimated air-sea CO_2_ fluxes from observations are mainly derived in six ways: the “top-down” approach^13^,^32^,^33^,^34^,^35^,^36^, the “bottom-up” approach^4^,^37^, the isotopic ratios of CO_2_ approach^38^, the combination of observed sea surface CO_2_ partial pressure and gas exchange coefficient^7^,^39^, an approach based on the relationship between the sea surface temperature and sea surface water partial pressure of CO_2_ (pCO_2_)^40^, and an observation-based neural network approach^5^,^41^. Rödenbeck *et al*.^42^ investigated various observation-based estimates from Surface Ocean pCO_2_ Mapping intercomparison (SOCOM), and found both of the consistencies and discrepancies. Most of the estimates are available after 1990 and have relatively short time durations.

Since the model years are not directly comparable to the real world counterpart, due to internal variability, the air-sea CO_2_ flux data with longer time durations are more suitable for evaluating and constraining the models simulations, and a continuous spatial distribution of the air-sea CO_2_ fluxes is also needed to estimate the carbon sink/source in regional oceans. Here, we choose an observation-based data set by using the “bottom-up” approach^4^ (http://apdrc.soest.hawaii.edu/datadoc/co2_flux.php). This approach uses a large amount of pCO_2_ observations to constrain a process model and produces monthly data from 1980 with a spatial resolution of 1° × 1°. As mentioned above, this observation-based data also has uncertainties, which mainly come from the limited key processes in the model, the potential errors in the important variables and the sensitive depth for correction in the assimilation system^4^. Although uncertainties exist, this observationally constrained data remains a good choice for evaluating the model performance due to its quantitative consistency with other estimated data and improvements in the temporal length and the resolution. This data have also been chosen to validate the air-sea CO_2_ fluxes in the CMIP5 models^43^.

## Methods and Results

We analyzed the air-sea CO_2_ fluxes simulated by the 13 CMIP5 models that provide both “esmhistorical” simulations and “esmrcp85” projection simulations.

In addition to the global ocean, three important regional oceans – the equatorial Pacific Ocean, North Atlantic Ocean and Southern Ocean – are also analyzed independently. As shown in the observed spatial distribution of the air-sea CO_2_ fluxes, the largest atmospheric CO_2_ source region is the equatorial Pacific Ocean, which is located between 30°S and 10°N and releases approximately 40% of the total CO_2_ emissions from the global ocean. In contrast, the North Atlantic Ocean, north of 40°N, acts as a significant atmospheric carbon sink, due to the cold temperatures and formation of North Atlantic Deep Water (NADW). The net fluxes composed of the absorption and emission south of 40°S are considered as the air-sea CO_2_ fluxes in Southern Ocean, which overall acts as a large atmospheric CO_2_ sink.

**Figure 1:**
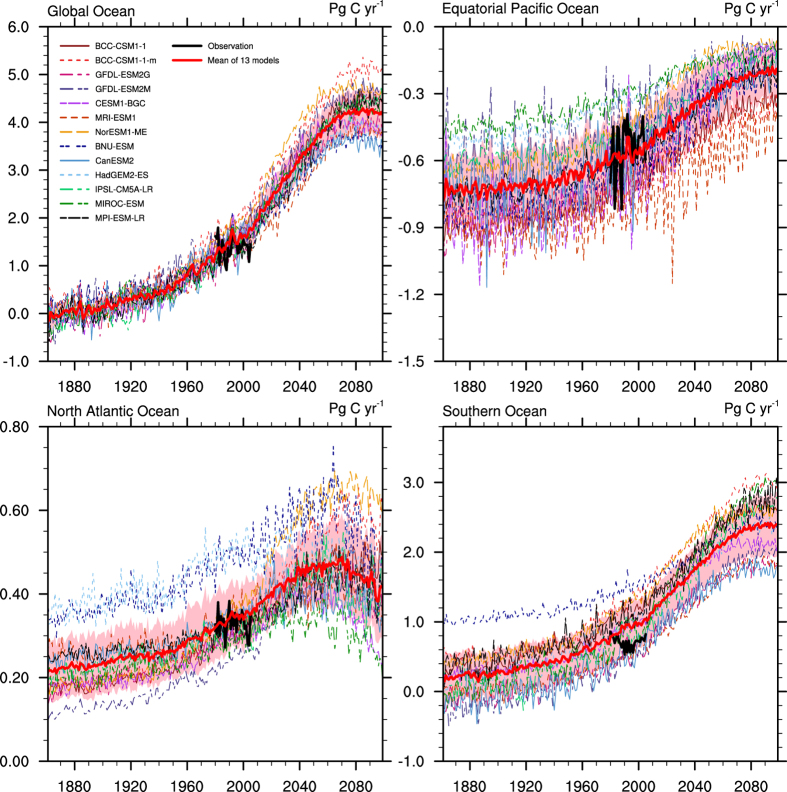
Historical (1861–2005) and projected (2006–2099) total air-sea CO_2_ fluxes simulated by 13 CMIP5 models in the global ocean and three regional oceans (equatorial Pacific Ocean, North Atlantic Ocean and Southern Ocean). The thick black line shows the observation-based estimates from 1980 to 2005, and the thick red line represents the multi-model ensemble mean. The pink shades represent the 1σ uncertainty range. Positive values mean the oceans are taking up atmospheric CO_2_. (Units: PgC/yr).

As shown in Fig. 1, a large spread in the simulated total air-sea CO_2_ fluxes remains in both the historical (1861–2005) and future (2006–2099) projected periods. The exact fluxes averaged over the observational period of 1980–2005 and projected periods are listed in Table 2, in addition to their 1σ uncertainty ranges. The relative largest uncertainties in the historical and future periods appear in the Southern Ocean and the equatorial Pacific Ocean, respectively.

In comparison with the observed fluxes (Table 2), the multi-model ensemble mean overestimates the global ocean and Southern Ocean mean carbon uptake from 1980 to 2005 by approximately 0.13 PgC/yr and 0.2 PgC/yr, respectively, while the mean state of the air-sea CO_2_ fluxes in the equatorial Pacific and North Atlantic Ocean simulated by the models are generally consistent with the observation-based estimates. Note that the observation-based carbon uptake in the Southern Ocean exhibits a decreasing trend before 1996, but most of the models are unable to capture these characteristics. The overestimate of the global ocean carbon uptake may in part be because the models ignore the pre-industrial river input of carbon and start their historical simulations from a level of nearly no air-sea CO_2_ fluxes, thereby they underestimating the oceanic emission of natural CO_2_.

As projected by the multi-model ensemble mean, the global ocean carbon sink will stop increasing at approximately 2070 after a period of continuous increase. The equatorial Pacific Ocean was a stable atmospheric carbon source (emitting approximately 0.72 PgC/yr) before the atmospheric CO_2_ concentration and global mean air temperature started to increase rapidly in approximately 1950. Subsequently, its release appears to decrease, and the decreasing rate is predicted to slow after approximately 2070. The carbon uptake in the North Atlantic Ocean is predicted to continuously increase until 2040, remain stable between approximately 2040 and 2070, then rapidly decrease after 2070. The changes in the air-sea CO_2_ fluxes in the Southern Ocean are similar to those of the global ocean, which will stop increasing by approximately 2070.

**Figure 2:**
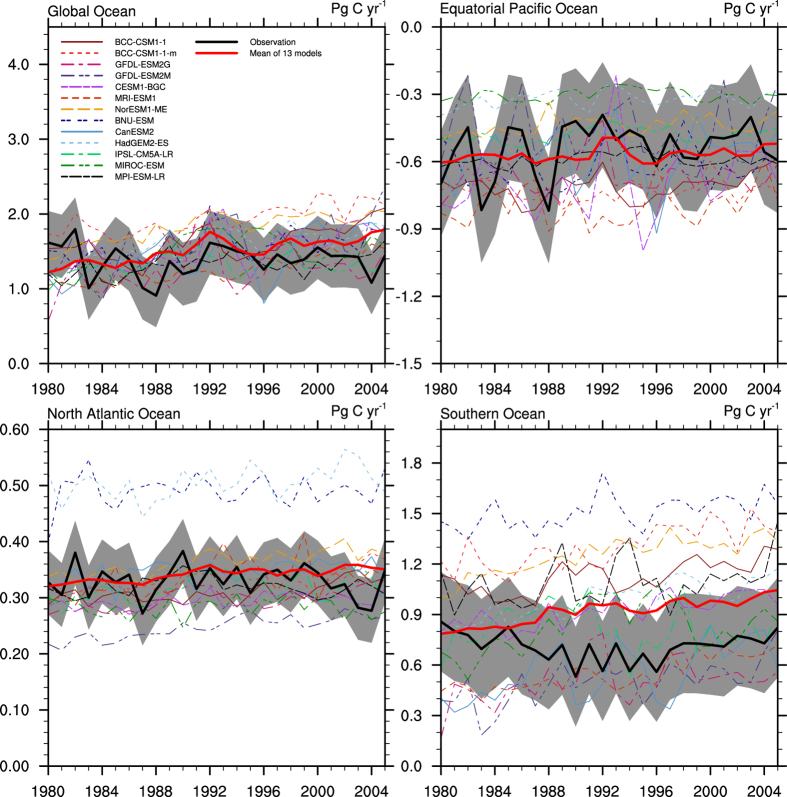
Historical air-sea CO_2_ fluxes from 1980 to 2005 simulated by 13 CMIP5 models for the global ocean and the three regional oceans (equatorial Pacific Ocean, North Atlantic Ocean and Southern Ocean). The thick black line shows the observation-based estimates, and the thick red line represents the multi-model ensemble mean. The grey shades represent the selection range. Positive values mean the oceans are taking up atmospheric CO_2_. (Units: PgC/yr).

One approach for narrowing the large spread in the projected air-sea CO_2_ fluxes is through model selection based on the ability of a model to reproduce the observed air-sea CO_2_ fluxes. Using the selected models based on the evaluation of their performance in the historical stage to project future changes is an effective way to reduce the spread in future projections. This process is also used in the projection of other variables^44^,^45^,^46^,^47^. The ability of the models to accurately simulate the mean stage, trend and variability of the air-sea CO_2_ fluxes affects the future projections. Here, we use a selection method that considers the three aspects of the simulated air-sea CO_2_ fluxes. We first calculate the standard deviation (σ) of the observation-based yearly air-sea CO_2_ fluxes from 1980 to 2005 and determine a selection range by adding and subtracting several σ values to the observation-based estimates. A model will be determined to be “good” at simulating the air-sea CO_2_ fluxes if more than 80% of the yearly points fall within the selected range. To determine the range, the σ is increased by 0.5 times repeatedly until at least 4 models are selected. The selection ranges are shown as the grey shades in Fig. 2. The exact multiples of σ used to determine the selection range and the selected models for the global ocean and the three regional oceans (equatorial Pacific Ocean, North Atlantic Ocean and Southern Ocean) are listed in Table 3. According to the selecting process, 6 models are selected based on their ability to reproduce the air-sea CO_2_ fluxes in the global ocean, and 6 models, 6 models, and 4 models are selected to simulate the air-sea CO_2_ fluxes in the equatorial Pacific Ocean, North Atlantic Ocean and Southern Ocean, respectively.

**Figure 3:**
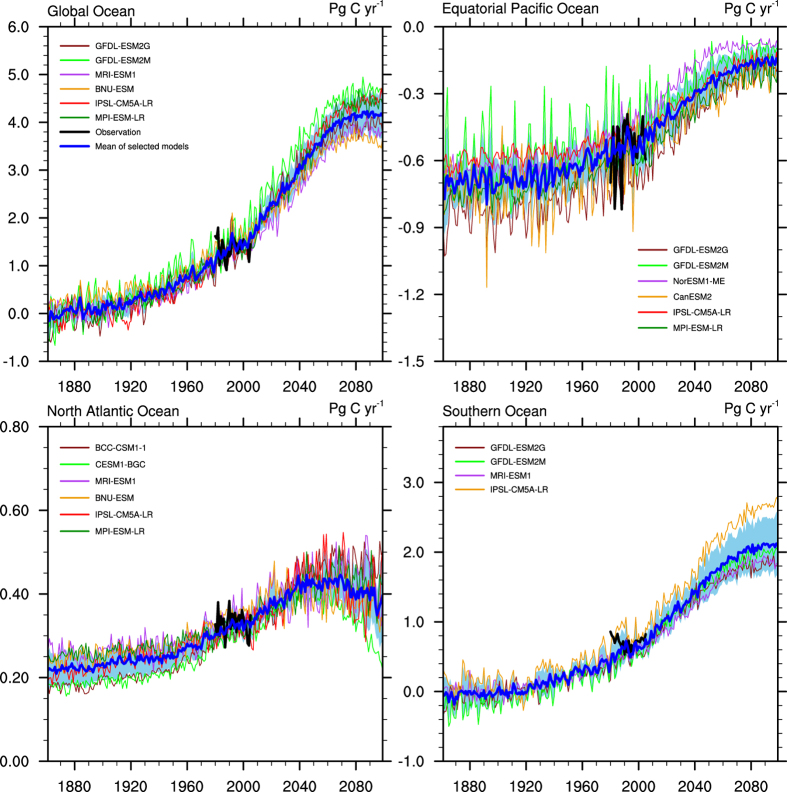
Historical (1861–2005) and projected (2006–2099) total air-sea CO_2_ fluxes simulated by selected CMIP5 models for the global ocean and three regional oceans (equatorial Pacific Ocean, North Atlantic Ocean and Southern Ocean). The thick black line shows the observation-based estimates from 1980 to 2005, and the thick blue line represents the multi-model ensemble mean of the selected models. The light blue shades represent the 1σ uncertainty range of the selected models. Positive values mean the oceans are taking up atmospheric CO_2_. (Units: PgC/yr).

As shown in Fig. 3, the air-sea CO_2_ fluxes simulated by the multi-model ensemble mean of the selected models are undoubtedly in better agreement with the observation-based estimates than those simulated by the ensemble mean of all 13 models. The differences in the mean air-sea CO2 fluxes during 1980–2005 between the simulations and observation-based estimates are reduced to within 0.01 PgC/yr for the global ocean, equatorial Pacific Ocean and North Atlantic Ocean and to 0.12 PgC/yr in the Southern Ocean. Additionally, the uncertainty ranges of the estimations are narrowed in the simulation of both the historical and projected air-sea CO_2_ fluxes. The characteristics of the evolution of the air-sea CO_2_ fluxes projected by the selected models are basically similar to those projected by all 13 models, but they differ in the specific values. The global ocean carbon uptake at the end of the 21^st^ century is projected to be 3.68–4.57 PgC/yr. The air-sea CO_2_ fluxes in the North Atlantic Ocean, equatorial Pacific Ocean and Southern Ocean are projected by the selected models to be 0.29–0.51 PgC/yr, −0.20–0.10 PgC/yr and 1.65–2.55 PgC/yr, respectively, at the end of the 21^st^ century. Although a range of projected uncertainty still exists, especially in the late 21^st^ century in the North Atlantic Ocean and the equatorial Pacific Ocean, the uncertainty ranges are effectively reduced.

Considering that the observation-based product we use has an uncertainty attached to it, we also do this analysis using another product of Park *et al*.^40^, which extrapolates seasonal relationship between the sea surface temperature and partial pressure of CO_2_ to the interannual time scale and is from the year of 1982. We find our results are almost independent of the choice of these two datasets, with the projection of 3.68–4.50 PgC/yr at the end of 21^st^ century by using Park *et al*.^40^ and 3.68–4.57 PgC/yr by using Valsala and Maksyutov^4^. The analysis using the product of Park *et al*. gives us additional confidence in our results.

The other approach for narrowing the large spread of the projected total air-sea CO_2_ fluxes is though constrained estimation based on the relationship between the historical and future air-sea CO_2_ fluxes derived from the model spread (hereafter referred to as “constrained estimation”). In this approach, the relationship between the historical and future air-sea CO_2_ flux conditions is first calculated for the 13 CMIP5 models, and the obtained relationship is then applied to the observation-based estimates to constrain the spread of the projected air-sea CO_2_ fluxes during the 21^st^ century. The rationale of this method is based on the fact that the models with greater air-sea CO_2_ fluxes in the historical period tend to quickly absorb atmospheric CO_2_ in the 21^st^ century, whereas the models with lower air-sea CO_2_ fluxes in the historical period tend to retain lower absorption rates for a relatively long period. The constrained estimation method has also been used to reduce the large projected spread in sea ice extent and ice-free time by Liu *et al*.^45^.

**Figure 4:**
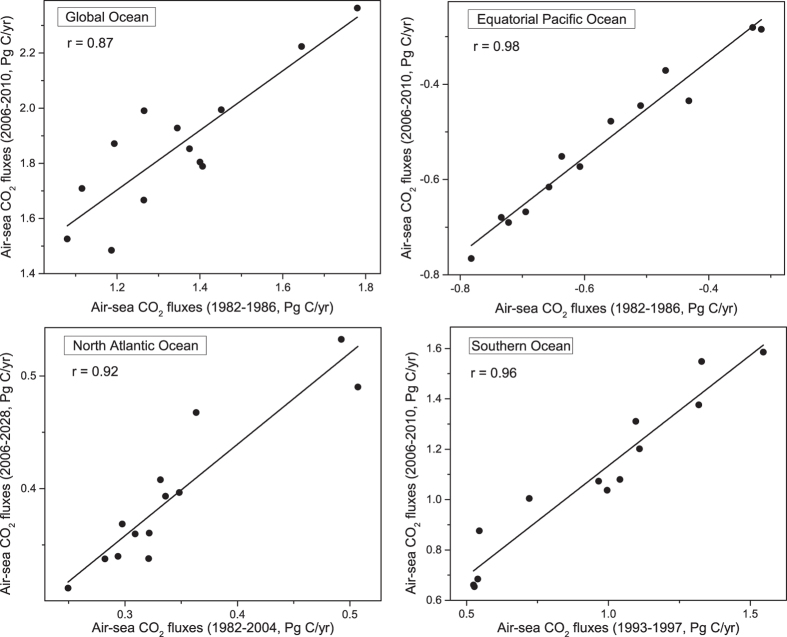
Scatter plot of air-sea CO_2_ fluxes in the historical mean state versus those in the first projected period state as simulated by the 13 CMIP5 models for the global ocean, equatorial Pacific Ocean, North Atlantic Ocean and Southern Ocean. The numbers in the upper-left corner of each graph are the correlation coefficients of the air-sea CO_2_ fluxes in the historical and projected period mean states.

Figure 4 shows the simulated air-sea CO_2_ fluxes averaged for a historical period versus the projected air-sea CO_2_ fluxes predicted by each of the 13 CMIP5 models averaged for a future period. Each dot represents a specific model. The correlation coefficients between the historical and future states are given in the upper-left corner of each graph. To represent the historical states of the different oceans, the 5-year period of 1982–1986 is chosen to represent the historical period of the global ocean and equatorial Pacific Ocean and the 23-year period of 1982–2004 and the 5-year period of 1993–1997 are chosen for the North Atlantic Ocean and Southern Ocean, respectively. These periods were chosen because the average correlation coefficients for air-sea CO_2_ fluxes in these periods and in every future period of the same length are the largest for the global ocean and the three regional oceans. Note that the means of the 5-year historical periods produce the most effective forecast for the global ocean, equatorial Pacific Ocean and Southern Ocean, while the temporal length of the most suitable period for projecting the air-sea CO_2_ fluxes in the North Atlantic Ocean is more than 20 years. The importance of the 5-year period for the equatorial Pacific Ocean and global ocean may be because the variability in the air-sea CO_2_ fluxes in the equatorial Pacific Ocean is dominated by the El Niño-Southern Oscillation (ENSO), which has a period of 2–7 years^40^,^48^,^49^, and because the variability in the global air-sea CO_2_ fluxes is governed by the variability in the air-sea CO_2_ fluxes in the equatorial Pacific Ocean^50^. The variability in the air-sea CO_2_ fluxes in the Southern Ocean may be driven by the Southern Annular Mode of climate variability^51^,^52^. Some researchers have noted that the changes in the air-sea CO_2_ fluxes in the North Atlantic Ocean may be associated with decadal variability in the North Atlantic Oscillation (NAO) and the Atlantic Multidecadal Oscillation (AMO)^53^,^54^, resulting in variability in the air-sea CO_2_ fluxes on a decadal time scale. Figure 4 shows the scatter plot of the historical period versus the first future period of the same length of time. These plots indicate that the projected air-sea CO_2_ fluxes are strongly associated with the simulated air-sea CO_2_ fluxes in the historical condition. The correlation coefficients all pass the 99% significance testing based on the assumption that each model is an independent sample, confirming that the historical state is a good predictor for the future state. For the global ocean and the three regional oceans, the regression functions between the historical and future states (the solid lines in Fig. 4) are calculated, and the observation-based estimates of the historical periods are then put into the regression functions to obtain the projected air-sea CO_2_ fluxes state in the corresponding period.

**Figure 5:**
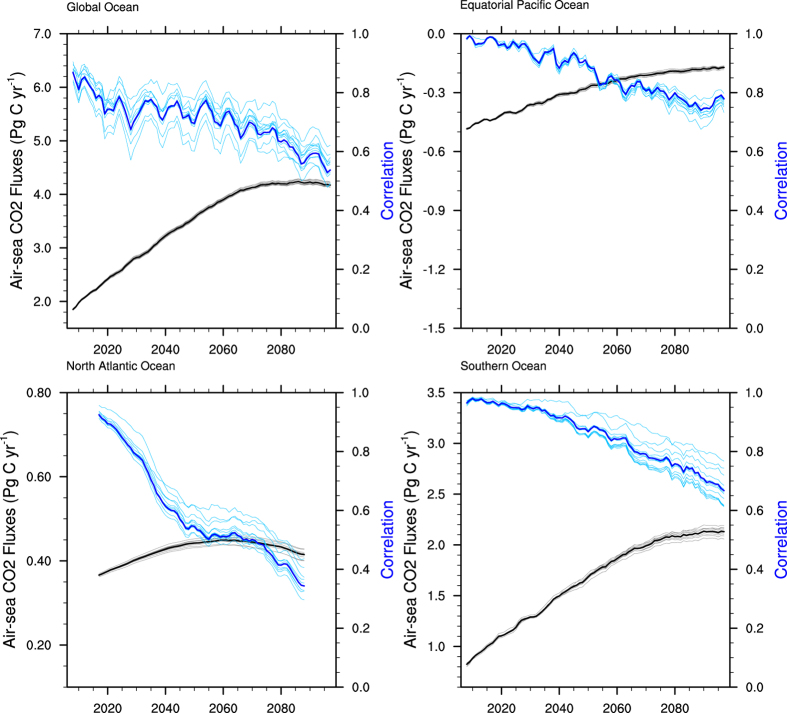
The evolution of the correlation coefficients (blue lines) and constrained estimation of air-sea CO_2_ fluxes (black lines) for the global ocean, equatorial Pacific Ocean, North Atlantic Ocean and Southern Ocean. The thick blue lines show the correlation coefficients of the mean state of every future period and the historical period for the 13 models, and the thick black lines show the constrained estimates based on using the observation-based estimates of the historical period to constrain the regression functions of the 13 models. The light blue lines and grey lines show the correlation coefficients and the constrained estimates calculated by repeating the same procedure for 12 of the 13 models (Jackknife method). This estimation is based on the relationship between the simulated air-sea CO_2_ fluxes averaged for a historical period and projected period.

Repeating the same constrained estimation process for every future period of the same length of time as the historical period, all the correlation coefficients (thick blue lines in Fig. 5) and projected fluxes (thick black lines in Fig. 5) are obtained. Taking the air-sea CO_2_ fluxes in the global ocean as an example, the average of the 5-year period of 1982–1986 is chosen as the historical state. Thus, the first 5-year period of 2006–2010 is centered on 2008, the second period of 2007–2011 is centered on 2009, and the last 5-year period of 2095–2099 is centered on 2097. The correlation coefficients are high at the beginning of the 21^st^ century and gradually decrease until the end of the 21^st^ century, which means that the credibility of the projections gradually decreases along with the weakening relationship between the historical state and the future state. All the calculated correlation coefficients for the equatorial Pacific Ocean and Southern Ocean pass the 95% significance testing. For the global ocean, except for the future periods centered on 2095, 2096 and 2097 (>90 significance), the other periods are closely associated with the historical state (>95 significance). For the North Atlantic Ocean, this method can be used to project the air-sea CO_2_ fluxes before the period centered on 2075 (>90 significance).

Because a specific model may unduly affect the correlation, we use the Jackknife method^55^ to estimate the uncertainty in the projection. The same procedure is repeatedly performed on the remaining 12 models after removing one model every time. Thus, we obtain the ensemble of 13 additional constrained estimates. The light blue lines in Fig. 5 show the correlations in this ensemble and the grey lines represent the constrained estimates based on the 12-model relationship. Although the correlations have a relatively evident spread, most of them pass the 90% significance test, and the 13 estimates show good agreement with the constrained estimate based on the 13 models.

**Figure 6:**
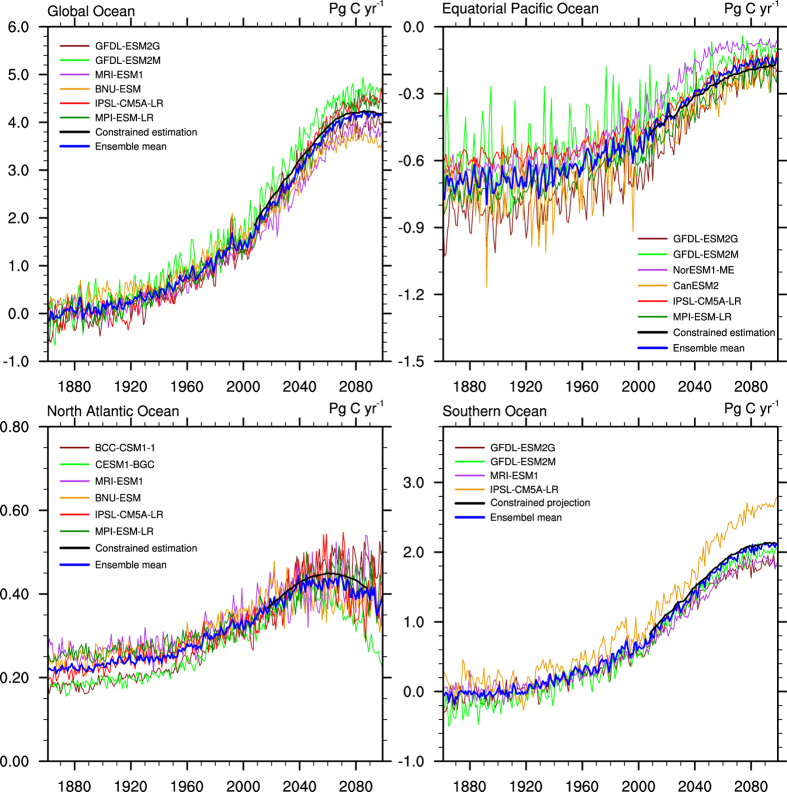
Historical (1861–2005) and projected (2006–2099) air-sea CO_2_ fluxes. The air-sea CO_2_ fluxes in the 21^st^ century are projected via the selected models method (thin colored lines represent the selected models and the thick blue lines show the multi-model ensemble mean of the selected models) and via the constrained estimation method (thick black lines) for the global ocean and the three regional oceans (equatorial Pacific Ocean, North Atlantic Ocean and Southern Ocean).

The projections based on the constrained estimation method are very similar to the projections based on the selected models method (Fig. 6), including the average state and evolution characteristics. This result further strengthens the projection on the air-sea CO_2_ fluxes. We also repeat the constrained analysis using the observation-based estimate of Park *et al*.^40^, and get the similar results.

## Conclusion and Discussion

Using model selection (removing the outlier models that fall outside a range of criteria based on the observation-based estimates) and constrained estimation (based on the close relationship between the simulated historical state and future state in the models), the spread projected by CMIP5 models in 21^st^ century is effectively reduced. Furthermore, the projections using the two different approaches exhibit strong agreement with each other. Compared to 3.70–4.69 PgC/yr projected by all the 13 models, the global ocean carbon uptake is projected to be 3.68–4.57 PgC/yr at the end of 21^st^ century in our study.

**Figure 7:**
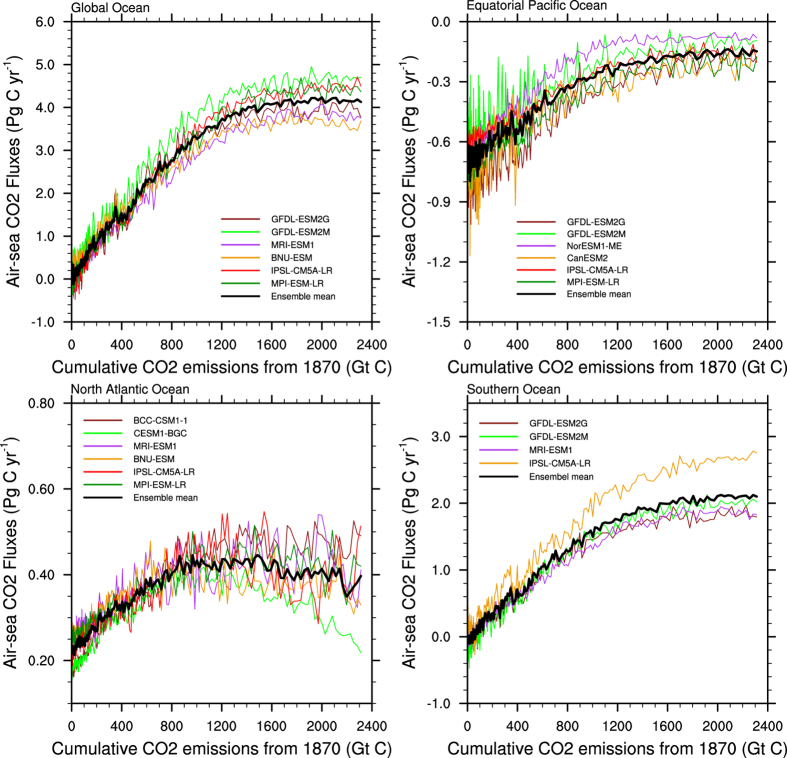
Air-sea CO_2_ fluxes in the global ocean, equatorial Pacific Ocean, North Atlantic Ocean and Southern Ocean versus the total global CO_2_ emissions since 1870 from CMIP5. The colored lines represent the selected models in the first approach, and the thick black lines show the ensemble mean of the selected models.

Based on the projections, the carbon sink capacity of the global ocean will increase with the rapid increase in atmospheric CO_2_ concentrations in the first half of the 21^st^ century and will then remain stable with no further growth after approximately 2070. This means that the ocean carbon sink will not always adapt to the growth of anthropogenic CO_2_ emissions and will stop increasing at a certain threshold of CO_2_ emissions, which will make the atmospheric CO_2_ concentration increase more rapidly when the ability of the ocean to absorb CO_2_ reaches a plateau. By reducing the model spread in projecting the air-sea CO_2_ fluxes, the changes in the future air-sea CO_2_ fluxes become more evident, and the turning point is independent at approximately 2070 when we apply the narrowing methods by using the observation-based data from no matter Park *et al*.^40^ or Valsala and Maksyutov^4^. Thus, we can use the selected models to analyze the critical cumulative emissions that affect the ocean carbon sink. The CO_2_ absorption of the global ocean will stop increasing when the cumulative CO_2_ emissions reach approximately 1,500 GtC (Fig. 7). The CO_2_ uptake of the Southern Ocean is projected to be similar to that of the global ocean, and the critical year of stagnant growth is also predicted to be approximately 2070, in association with a cumulative CO_2_ quantity of approximately 1,600 GtC. The atmospheric CO_2_ source in the equatorial Pacific Ocean is projected to decrease in the early 21^st^ century, which appears to decrease its positive contribution to atmospheric CO_2_. The emissions will stop decreasing in approximately 2075, indicating that the equatorial Pacific Ocean will not transform from a CO_2_ source into a CO_2_ sink. The carbon sink capacity of the North Atlantic Ocean will also increase during the early years of 21^st^ century, remain stable for approximately 20 years, then weaken in the late 21^st^ century. The absorption of North Atlantic Ocean will stop increasing and will start to decrease at the critical cumulative CO_2_ emissions of 800 GtC and 1500 GtC, respectively. The summarized findings in Fig. 7 suggest that the atmospheric CO_2_ concentration may increase more rapidly after the CO_2_ emissions reach 800–1600 GtC due to significant changes in the absorption abilities of the oceans.

The projections show that the CO_2_ absorption of the oceans will not continue to increase with the atmospheric CO_2_ concentration. Instead, they will remain stable or even decrease after the CO_2_ emissions reach a certain threshold, which means that increases in the atmospheric CO_2_ concentration may accelerate. The increasing CO_2_ concentration will result in global warming and more frequent climate extremes^56^,^57^. The cumulative anthropogenic CO_2_ emissions reached 515 PgC in 2011^3^; thus, stricter CO_2_ emission policies are needed to mitigate the rapid increase in atmospheric CO_2_ concentration to allow the ocean to absorb more CO_2_ in the future.

The limitation of our work is also needed to be addressed. The two methods used to narrow the spread in the model projections of air-sea CO_2_ fluxes are partly observation-based. The observation-based estimates are used to select “good” models in the first approach and to constrain the model-based relationship in the second approach. As mentioned in the data section, direct observations of the air-sea CO_2_ fluxes are very limited, and continuous data mainly come from data interpolation and assimilation approaches. So our projections contain the uncertainties from the observation-based estimates. We test our results using another observation-based product of Park *et al*.^40^, and get almost the same projection of the air-sea CO_2_ fluxes (3.68–4.50 PgC/yr). More products of air-sea CO_2_ fluxes with long time durations are needed to further estimate the projection uncertainties from the observation-based estimates in the future. Although uncertainties exist in the observation-based estimates, our approach provides two effective ways to reduce the spread of future projections of the air-sea CO_2_ fluxes and is helpful for producing more reliable model-based climate projections. The more reliable observation-based estimates as well as the in-depth understanding of carbon cycle process are the basis for improving the performance of the carbon cycle models. Broader and more continuous direct observations and improved reanalysis methods are needed to reduce the uncertainty in the air-sea CO_2_ flux data and further reduce the uncertainty in the future projection.

## Additional Information

**How to cite this article**: Wang, L. *et al*. Narrowing the spread in CMIP5 model projections of air-sea CO_2_ fluxes. *Sci. Rep.* **6**, 37548; doi: 10.1038/srep37548 (2016).

**Publisher’s note:** Springer Nature remains neutral with regard to jurisdictional claims in published maps and institutional affiliations.

## Figures and Tables

**Table 1:** Affiliations and ocean carbon components of the 13 CMIP5 models.

Sponsor, Country	CMIP5 model	Ocean carbon component
Beijing Climate Center (BCC), China	BCC-CSM1-1	Ocean Carbon-Cycle Model Intercomparison Project Phase2 (OCMIP-2)
	BCC-CSM1-1-m	Ocean Carbon-Cycle Model Intercomparison Project Phase2 (OCMIP-2)
Geophysical Fluid Dynamics Laboratory (GFDL), United States	GFDL-ESM2G	Tracers of Ocean Phytoplankton with Allometric Zooplankton (TOPAZ)
	GFDL-ESM2M	Tracers of Ocean Phytoplankton with Allometric Zooplankton (TOPAZ)
National Center for Atmospheric Research (NCAR), United States	CESM1-BGC	Biogeochemical Elemental Recycling (BEC)
Model for Interdisciplinary Research on Climate (MIROC), Japan	MRI-ESM1	Nutrients-Phytoplankton-zooplankton-detritus (NPZD)
Norwegian Climate Center (NCC), Norway	NorESM1-ME	Miami Isopycnic Coordinate Ocean Model (MICOM)
Beijing Normal University (BNU), China	BNU-ESM	Idealized ocean biogeochemistry (IBGC)
Canadian Centre for Climate Modelling and Analysis	CanESM2	Canadian Model of Ocean Carbon (CMOC)
Met Office (UKMO), UK	HadGEM2-ES	Diatom version of the Hadley Centre Ocean Carbon Cycle model (Diat-HadOCC)
L’Institut Pierre-Simon Laplace (IPSL), France	IPSL-CM5A-LR	Pelagic Interactive Scheme for Carbon and Ecosystem Studies (PISCES)
Model for Interdisciplinary Research on Climate (MIROC), Japan	MIROC-ESM	Nutrients-Phytoplankton-zooplankton-detritus (NPZD)
Max Planck Institute (MPI), Germany	MPI-ESM-LR	Hamburg Model of the Ocean Carbon Cycle (HAMOCC)

**Table 2:** Observation-based air-sea CO_2_ fluxes during 1980–2005 and the means of historical and future projected total air-sea CO_2_ fluxes simulated by 13 CMIP5 models.

Total air-sea CO_2_ fluxes (units: PgC/yr)	Observation-based estimates (1980–2005)	Historical mean (1980–2005)	Future projected mean (2080–2099)
Global Ocean	1.38	1.51 ± 0.16	4.22 ± 0.46
Equatorial Pacific Ocean	−0.54	−0.57 ± 0.16	−0.21 ± 0.12
North Atlantic Ocean	0.33	0.34 ± 0.08	0.43 ± 0.10
Southern Ocean	0.71	0.91 ± 0.33	2.38 ± 0.43

**Table 3:** The multiples of σ and selected models used to simulate the air-sea CO_2_ fluxes in the global ocean, equatorial Pacific Ocean, North Atlantic Ocean and Southern Ocean.

	Global Ocean	Equatorial Pacific Ocean	North Atlantic Ocean	Southern Ocean
Multiples of σ	2.0	2.0	2.0	3.5
Selected models	GFDL-ESM2G	GFDL-ESM2G	BCC-CSM1-1	GFDL-ESM2G
	GFDL-ESM2M	GFDL-ESM2M	CESM1-BGC	GFDL-ESM2M
	MRI-ESM1	NorESM1-ME	MRI-ESM1	MRI-ESM1
	BNU-ESM	CanESM2	BNU-ESM	IPSL-CM5A-LR
	IPSL-CM5A-LR	IPSL-CM5A-LR	IPSL-CM5A-LR	
	MPI-ESM-LR	MPI-ESM-LR	MPI-ESM-LR	
