# Corrigendum: Narrowing the spread in CMIP5 model projections of air-sea CO_2_ fluxes

**DOI:** 10.1038/srep43499

**Published:** 2017-04-10

**Authors:** Lei Wang, Jianbin Huang, Yong Luo, Zongci Zhao

Scientific Reports 6: Article number: 37548; 10.1038/srep37548 published online: 11 28 2016; updated: 04 10 2017.

The Acknowledgements section in this Article is incomplete.

“We acknowledge the World Climate Research Programme’s Working Group on Coupled Modeling, which is responsible for CMIP. We thank the climate groups (listed in Table 1) for providing the model output. We would also like to thank the anonymous reviewer for the comments and suggestions that greatly improved the original manuscript. Our work was completed on the “Explorer 100” cluster system at Tsinghua National Laboratory for Information Science and Technology.

should read:

“This work was supported by the project supported by the State Key Development Program for Basic Research of China (Grant No. 2013CB956604), the project supported by the National Science Foundation for Young Scientists of China(Grant No. 41305054), and the project supported by the State Key Development Program for Basic Research of China(Grant No. 2013CBA01805). We acknowledge the World Climate Research Programme’s Working Group on Coupled Modelling, which is responsible for CMIP. We thank the climate groups (listed in Table 1) for providing the model output. We would also like to thank the anonymous reviewer for the comments and suggestions that greatly improved the original manuscript. Our work was completed on the “Explorer 100” cluster system at Tsinghua National Laboratory for Information Science and Technology”.

